# Toll-Like Receptor Activation by Generalized Modules for Membrane Antigens from Lipid A Mutants of Salmonella enterica Serovars Typhimurium and Enteritidis

**DOI:** 10.1128/CVI.00023-16

**Published:** 2016-04-04

**Authors:** Omar Rossi, Mariaelena Caboni, Aurel Negrea, Francesca Necchi, Renzo Alfini, Francesca Micoli, Allan Saul, Calman A. MacLennan, Simona Rondini, Christiane Gerke

**Affiliations:** Novartis Vaccines Institute for Global Health, S.r.l., Siena, Italy

## Abstract

Invasive nontyphoidal Salmonella (iNTS) disease is a neglected disease with high mortality in children and HIV-positive individuals in sub-Saharan Africa, caused primarily by Africa-specific strains of Salmonella enterica serovars Typhimurium and Enteritidis. A vaccine using GMMA (generalized modules for membrane antigens) from *S.* Typhimurium and *S.* Enteritidis containing lipid A modifications to reduce potential *in vivo* reactogenicity is under development. GMMA with penta-acylated lipid A showed the greatest reduction in the level of cytokine release from human peripheral blood monocytes from that for GMMA with wild-type lipid A. Deletion of the lipid A modification genes *msbB* and *pagP* was required to achieve pure penta-acylation. Interestingly, Δ*msbB* Δ*pagP* GMMA from *S.* Enteritidis had a slightly higher stimulatory potential than those from *S.* Typhimurium, a finding consistent with the higher lipopolysaccharide (LPS) content and Toll-like receptor 2 (TLR2) stimulatory potential of the former. Also, TLR5 ligand flagellin was found in Salmonella GMMA. No relevant contribution to the stimulatory potential of GMMA was detected even when the flagellin protein FliC from *S.* Typhimurium was added at a concentration as high as 10% of total protein, suggesting that flagellin impurities are not a major factor for GMMA-mediated immune stimulation. Overall, the stimulatory potential of *S.* Typhimurium and *S.* Enteritidis Δ*msbB* Δ*pagP* GMMA was close to that of Shigella *sonnei* GMMA, which are currently in phase I clinical trials.

## INTRODUCTION

Nontyphoidal salmonellae (NTS) have recently been recognized as a common cause of bloodstream infections in sub-Saharan Africa. The majority of the invasive cases are associated with Salmonella enterica serovars Enteritidis and Typhimurium (1, 2). Invasive NTS (iNTS) disease occurs predominantly in young children, children with malnutrition or malaria, and individuals of all ages with underlying conditions, such as HIV (1–3). In children, the estimated disease incidence is approximately 500 per 100,000 per year (4). In adults, 95% of all cases are HIV associated (1), with an incidence of approximately 2,000 per 100,000 HIV-positive individuals per year (1–3). iNTS disease is associated with a high mortality rate, estimated to be as high as 20%. No vaccines against iNTS disease are currently available for use in humans. Multiple approaches, predominantly using live-attenuated strains or O antigen polysaccharide conjugated to carrier proteins, are under development (5–7).

Gram-negative bacteria naturally shed outer membrane blebs, called native outer membrane vesicles (NOMV), that are composed of outer membrane lipids, outer membrane proteins, and periplasmic proteins and that present surface antigens in their natural conformations and contexts (8). NOMV have been proposed as a vaccine (8). Immunization with *S.* Typhimurium NOMV protected mice against challenge with homologous bacteria (9). However, NOMV are generally present in low quantities. We have recently developed a high-yield production method and corresponding industrial processes for outer membrane blebs by using genetic modification of bacteria to enhance the shedding of blebs (10–12). The genetically derived blebs were called generalized modules for membrane antigens (GMMA; from the Italian word *gemma*, meaning “bud”). The GMMA process allows manufacturing at a low cost of goods, making GMMA attractive vaccine candidates, particularly suited for the development of vaccines for low- and middle-income countries.

As part of a program to develop a vaccine against iNTS disease (13, 14), we have applied this technology to *S.* Typhimurium and *S.* Enteritidis. As with the induction of hyperblebbing in Shigella spp. (10, 12), the required genetic modification for Salmonella was the deletion of the *tolR* gene (15), which is involved in the linkage of the inner and outer bacterial membranes. Outer membrane blebs are derived from the surfaces of Gram-negative bacteria and thus contain potent immunostimulatory components, especially lipopolysaccharide (LPS) and lipoproteins, which are recognized by Toll-like receptor 4 (TLR4) and TLR2, respectively (16). In addition, outer membrane blebs from flagellated bacteria have been shown to contain flagellin (17, 18), the ligand for TLR5 (19). While the presence of these stimulatory molecules likely contributes to their high immunogenicity (10, 11), it also potentially gives rise to reactogenicity.

Reducing reactogenicity while maintaining immunogenicity is an important step in GMMA vaccine development. Our focus has been on modifying lipid A, the endotoxic part of LPS (11, 20), along the lines of the preparation of a Neisseria meningitidis NOMV vaccine candidate from an *lpxL1* mutant (Neisseria group B) that was shown to be safe and immunogenic in humans (21). Recently, we showed that Shigella and Neisseria GMMA with penta-acylated lipid A had a substantially reduced ability to stimulate cytokine release from human peripheral blood monocytes (PBMC) (11, 12, 20). GMMA from an S. sonnei Δ*htrB* mutant are currently being tested in a phase I trial (12).

In Salmonella, the situation is more complex, due to the presence of additional lipid A-modifying enzymes and flagellin. The classical hexa-acylated lipid A, which is shared by Salmonella, Escherichia coli, and Shigella spp., is the most endotoxic lipid A structure (22). In contrast to Shigella spp., Salmonella and E. coli also generate hepta-acylated lipid A through the transfer of an additional palmitoyl (C_16:0_) chain in the secondary position on the hydroxymyristate chain at position 2 of lipid A, catalyzed by PagP under certain environmental conditions (23). Several approaches have been taken to reduce the endotoxicity of Salmonella LPS. Among these, genetic approaches have been used to modify the acyl chain composition (24–27) through inactivation of the late acyl transferases HtrB (LpxL, WaaM) (24) and MsbB (LpxM, WaaN) (27), which transfer secondary lauroyl (C_12:0_) and myristoyl (C_14:0_) chains in positions 3′ and 2′, respectively (see Fig. S1 in the supplemental material).

Inactivation of *msbB* in *S.* Typhimurium results in a mixed lipid A population containing penta- and hexa-acylated species lacking the myristoyl chain, which is present in the wild-type (WT) hexa- and hepta-acylated lipid A forms (25–27). LPS and heat-killed bacteria from the mutant strains stimulate less cytokine release from human and murine cells than the respective WT LPS or bacteria (25–27). The additional deletion of *pagP* results in a purely penta-acylated lipid A population with further reduced stimulatory potential (26). Deletion of the *htrB* gene in *S.* Typhimurium has pleiotropic effects on the lipid A structure, including replacement of the lauroyl chain with a palmitoleoyl chain (24), which has also been observed in *htrB* mutants of E. coli and Shigella flexneri 2a (20, 28). The resulting LPS elicited less tumor necrosis factor alpha (TNF-α) secretion from a mouse macrophage cell line than LPS from WT *S.* Typhimurium (29).

Furthermore, in addition to lipidated membrane proteins that are agonists for TLR2 (16), Salmonella GMMA may contain TLR5-activating flagellin protein as an impurity (19). Flagellin is the major repeating structural unit protein of the bacterial flagellar motility apparatus. Flagellin has been shown to stimulate the production of cytokines, including TNF-α and interleukin 6 (IL-6), from various human cell lines (30, 31) and is thought to contribute to septic shock caused by flagellated Gram-negative bacteria (32).

In this study, we assess the contributions of different lipid A modifications and flagellin impurities in GMMA from *S.* Typhimurium and *S.* Enteritidis to *in vitro* stimulation of human PBMC with the aim of selecting an approach for further iNTS GMMA vaccine development for use in humans. We demonstrate that the combined deletion of *msbB* and *pagP* decreases the ability of GMMA to stimulate IL-6 release from PBMC by approximately 200-fold in *S.* Typhimurium GMMA and approximately 30-fold in *S.* Enteritidis GMMA, to levels similar to that of a Shigella sonnei GMMA vaccine candidate currently in phase I clinical trials. We further show that monomeric flagellin impurities, specifically FliC from *S.* Typhimurium strain ATCC 14028, at levels as high as 10% of total GMMA protein do not contribute significantly to GMMA-mediated stimulation of PBMC *in vitro*.

## MATERIALS AND METHODS

### Strains and mutant generation.

Salmonella enterica serovar Typhimurium isolate 1418 (LT-2 collection [33]; University of Calgary) and Salmonella enterica serovar Enteritidis 618 (CEESA EASSA collection II [34]; Quotient Bioresearch Limited), both isolated from animals, were chosen as parent strains on the basis of a screen performed previously (35). The Salmonella mutant strains used in this study and their abbreviated names are listed in Table 1. To generate the mutants, the kanamycin resistance gene *aph* was used to replace the *tolR* gene, the chloramphenicol resistance gene *cat* was used to replace the *htrB* and *pagP* coding sequences, and the tetracycline resistance cassette *tetRA* was used to replace the *msbB* gene. The desired resistance cassette replacement constructs were amplified using forward and reverse primers composed of approximately 50 bp homologous to the flanking regions of the gene to be deleted and approximately 20 bp at the 3′ end matching the flanking region of the respective resistance gene. The primers were designed to be suitable for both *S.* Typhimurium and *S.* Enteritidis and are listed in Table 2. The PCR products were purified and were used to transform recombination-prone Salmonella recipient cells by following methods described previously (10, 36).

**TABLE 1:** Strains used in this study and their abbreviations

Strain name abbreviation	Genotype
STm_G_	*S.* Typhimurium Δ*tolR*::*aph*
STm_G_ Δ*htrB*	*S.* Typhimurium Δ*tolR*::*aph* Δ*htrB*::*cat*
STm_G_ Δ*msbB*	*S.* Typhimurium Δ*tolR*::*aph* Δ*msbB*::*tetRA*
STm_G_ Δ*msbB* Δ*pagP*	*S.* Typhimurium Δ*tolR*::*aph* Δ*msbB*::*tetRA* Δ*pagP*::*cat*
SEn_G_	*S.* Enteritidis Δ*tolR*::*aph*
SEn_G_ Δ*htrB*	*S.* Enteritidis Δ*tolR*::*aph* Δ*htrB*::*cat*
SEn_G_ Δ*msbB*	*S.* Enteritidis Δ*tolR*::*aph* Δ*msbB*::*tetRA*
SEn_G_ Δ*msbB* Δ*pagP*	*S.* Enteritidis Δ*tolR*::*aph* Δ*msbB*::*tetRA* Δ*pagP*::*cat*
SEn_G_ Δ*msbB* Δ*htrB*	*S.* Enteritidis Δ*tolR*::*aph* Δ*msbB*::*tetRA* Δ*htrB*::*cat*

**TABLE 2:** List of primers used in this study

Primer name	Sequence (5′ → 3′)
*tolR* for	CCAGGCGTTTACCGTAAGCGAAAGCAACAAGGGGTAAGCCCTCTGGTAAGGTTGGGAA
*tolR* rev	CCTGTTACTCGCCGTCTTTCAAGCCAACGGGACGCAGACTTCAGAAGAACTCGTCAAG
*msbB* for	AGGTAGTACAGGGTTTGTCAGCATAAAGCCTCTCTTACGAGAGGCTTTATTTAAGACCCACTTTCACATT
*msbB* rev	AGACGTCGCTACACTATTCACAATTCCTTTTCGCGTCAGCAGACCCTAAGCACTTGTCTCCTG
*pagP* for	GGAGCGCGTGACGGTTCTGAGTGCTAAATCAAACGCCGTTAACCCGATGTGTAGGCTGGAGCTGCTTCG
*pagP* rev	GTACAACAATTGTGATGCATTTTGTCCAGTCGAACTTTGCGAAAAAGTGATACATATGAATATCCTCCTTAG
*htrB* for	CAAAAAGATGCGAGAATACGGGGAATTGTTCGTTGAAAGACAGGATAGAAGTGTAGGCTGGAGCTGCTTCG
*htrB* rev	CTTTTAAAGCTAAAAGAGGGGAAAAATTGCAGCCTGACGGCTGCAATCCTGCATATGAATATCCTCCTTAG

### GMMA production and purification.

Bacterial strains were routinely grown at 30°C in liquid or on solid Luria-Bertani medium without salt (LBON). For GMMA production, overnight cultures were grown in the presence of selective antibiotics, i.e., kanamycin (30 μg/ml), chloramphenicol (20 μg/ml), or tetracycline (20 μg/ml), and were used to inoculate the production medium (without antibiotics) to an optical density (OD) at 600 nm of 0.03 to 0.05. Production cultures were incubated at 30°C and 200 rpm overnight. Culture supernatants were collected by centrifugation for 10 min at 5,000 × *g*, followed by 0.22-μm filtration. GMMA were concentrated using an Amicon stirrer cell with a regenerated cellulose filter with a nominal molecular size limit of 100 kDa (Amicon Ultracell) under a nitrogen flow. The retentate was collected in 70-ml propylene ultracentrifuge tubes (Beckman Coulter) and was ultracentrifuged at 186,000 × *g* for 2 h at 4°C using a 45 Ti rotor (Beckman Coulter). Pellets were resuspended in 2 ml of phosphate-buffered saline (PBS), followed by 0.22-μm filtration. GMMA were stored at 4°C.

### Quantification of GMMA protein and KDO.

GMMA quantities are expressed as the total protein present in GMMA quantified using the DC protein assay (Bio-Rad), which is based on the Lowry assay (37). Core reducing end KDO (2-keto-3-deoxy-octonate), obtained after lipid A cleavage, was quantified using the semicarbazide/high-performance liquid chromatography–size exclusion chromatography (HPLC-SEC) method (38) as adapted previously for use in GMMA (20).

### Negative staining and transmission electron microscopy.

GMMA were adsorbed to Formvar/carbon-coated grids, negatively stained with uranyl acetate as described previously (20), and subsequently observed with a Tecnai G2 Spirit transmission electron microscope (FEI, Eindhoven, The Netherlands) operating at 80 kV. Electron micrographs were recorded at a nominal magnification of ×87,000. GMMA diameters were measured manually in comparison with the scale bar.

### MALDI-TOF analysis of lipid A.

Lipid A was precipitated from GMMA using mild-acid hydrolysis and was analyzed with an Ultraflex matrix-assisted laser desorption ionization–time of flight (MALDI-TOF) mass spectrometer (Bruker Daltonics) in negative-ion reflectron mode as reported previously (20). A peptide calibration standard (Bruker Daltonics) was included in each analysis. The samples and the standard were mixed with Super DHB solution (Sigma-Aldrich), a matrix substance for MALDI-TOF analyses. The *m/z* ratios were determined by flexAnalysis software in comparison to the peptide standard.

### PBMC isolation and stimulation (monocyte activation test [MAT]).

Buffy coats from four different donors were used to isolate PBMC by Ficoll density centrifugation as reported previously (39). PBMC were stimulated at a density of 2 × 10^5^/well in RPMI 1640 medium supplemented with 25 mM HEPES, 2 mM glutamine, 10% fetal bovine serum (FBS), and 1% penicillin-streptomycin solution (Pen-Strep; Invitrogen) in 96-well round-bottom plates with 0.0001 to 1,000 ng of GMMA/ml in 10-fold steps (20). The amount of IL-6 released by PBMC exposed to GMMA at the lowest concentration (0.0001 ng/ml) was similar to that released by PBMC exposed to PBS as a control (data [not shown] similar to those in reference 20) and thus was used as the baseline in the experiments. LPS from E. coli R515 (TLRgrade; Enzo Life Sciences) and, in some experiments, FliC from *S.* Typhimurium strain ATCC 14028 (Adipogen) were used as controls at 0.0001 to 1,000 ng/ml in 10-fold steps. Cells were incubated for 4 h at 37°C, and supernatants were recovered after centrifugation at 400 × *g* and were stored at −80°C until analysis.

### Cytokine analysis by ELISA and comparison of stimulatory potentials of GMMA.

IL-6 released into the supernatants was quantified by enzyme-linked immunosorbent assays (ELISA) using a human IL-6 capture antibody (catalog no. 14-7069; eBioscience) and biotin-conjugated anti-human IL-6 (catalog no. 13-7068; eBioscience) as the detection antibody as reported previously (20). IL-6 concentrations in the samples were calculated in comparison to a standard prepared with recombinant human IL-6 (catalog no. 39-8069; eBioscience). Results below the detection limit were assigned a value equivalent to half of the detection limit. The IL-6 levels of the samples were plotted against the GMMA concentration. The stimulatory potentials of different GMMA were compared using the GMMA concentrations needed to obtain a 10-fold increase in the amount of IL-6 released over the average level obtained at the lowest GMMA concentration (background level). The 10-fold increase over the baseline was chosen because this threshold is in the middle of the linear part of the sigmoidal curves and in line with previous analyses of GMMA (20, 40).

### TLR-blocking experiments.

In TLR-blocking experiments in the MAT, 15 μg/ml of a TLR2-blocking antibody (catalog no. 14-9024-82; eBioscience), 25 μg/ml of a TLR4-blocking antibody (catalog no. 16-9917-82; eBioscience), or 10 μg/ml of a TLR5-blocking antibody (catalog no. maba2-htlr5; InvivoGen) (all final concentrations in the assay) was added to the PBMC and was incubated for 30 min before the addition of GMMA. Subsequently, the MAT and IL-6 quantification were carried out as described above. All blocking antibodies were confirmed to be functional by demonstrating that preincubation of PBMC with these antibodies inhibited the induction of IL-6 release to <10% of that with nonpreincubated PBMC when TLR-specific ligands were used as stimuli. The experiments were performed with two different concentrations of the specific ligands Pam3CSK4 (catalog no. tlrl-pms; InvivoGen) for TLR2-specific activation, LPS (from E. coli R515; TLRgrade; Enzo Life Sciences) for TLR4-specific activation, and FliC (from *S.* Typhimurium strain ATCC 14028; Adipogen) for TLR5-specific activation, which resulted in IL-6 release in the linear part of the sigmoidal response curve with nonpreincubated PBMC. For statistical analysis of the blocking results, the ratio of the amount of IL-6 produced by PBMC treated with anti-TLR2, anti-TLR4, or anti-TLR5 to the amount of IL-6 produced by PBMC not treated with blocking antibodies and stimulated with the same concentration of GMMA was calculated in order to normalize the results of the TLR-blocking experiments using PBMC from different donors. The ratio was determined for each replicate in the experiments. To visualize the order of magnitude of IL-6 release and compare the stimulatory potentials of the different types and concentrations of GMMA, the average ratios for the blocking experiments were then multiplied by the mean IL-6 release by PBMC that were not treated with blocking antibodies and were stimulated with the specific concentration of GMMA.

### TLR-specific assays.

Human embryonic kidney 293 (HEK293) cells expressing luciferase under the control of the NF-κB promoter and stably transfected with either human TLR5 (HEK293-TLR5 cells), TLR4, MD2, and CD14 (HEK293-TLR4 cells), or TLR2 (HEK293-TLR2 cells) were maintained in Dulbecco's modified Eagle medium (DMEM) supplemented with 4.5 g/liter of glucose, 25 mM HEPES, 10% FBS, 1% Pen-Strep, and specific antibiotics for the different cell lines: puromycin (5 μg/ml), blasticidin (10 μg/ml), and hygromycin (250 μg/ml) for HEK293-TLR4 cells, blasticidin and hygromycin for HEK293-TLR5 cells, and puromycin and hygromycin for HEK293-TLR2 cells. A total of 25,000 cells/well were seeded in 90 μl of complete DMEM without specific antibiotics in 96-well microclear luciferase plates (PBI International) and were incubated for 24 h at 37°C. Ten microliters of serial 10-fold dilutions of GMMA in PBS (final concentration in the assay, 0.0001 to 1,000 ng/ml) was added. In the TLR-5-specific assay, FliC from *S.* Typhimurium strain ATCC 14028 (Adipogen) was used as the standard and was tested at the same concentrations as GMMA (10-fold dilution series in PBS with 0.0001 to 1,000 ng FliC/ml in the assay). After incubation for 5 h at 37°C, supernatants were removed, and cells were lysed for 20 min at room temperature using 20 μl/well of 1:5-diluted “passive lysis buffer” (Promega). The luciferase produced was detected using 100 μl/well of luciferase assay reagent (Promega), and the light emitted was immediately quantified using an Lmax II^384^ luminometer (Molecular Devices). NF-κB activation of cells stimulated with GMMA is expressed as the fold increase in emitted light over the average result for PBS-stimulated control cells. For comparison of the TLR-specific activities of different samples, the sample concentrations needed to obtain 10-fold (TLR2) or 3-fold (TLR4) NF-κB induction were used as described previously (20). TLR5-specific activities were compared using the sample concentration needed for 10-fold NF-κB induction. All thresholds were chosen based on the fact that they were in the middle of the linear part of the sigmoidal curves.

### Flagellin quantification (monomeric flagellin).

The concentration of flagellin in GMMA was determined using the TLR5-specific assay in combination with a standard generated with purified FliC from *S.* Typhimurium strain ATCC 14028 (Adipogen; catalogue no. AG-40B-0025-C010). Flagellin concentrations in GMMA samples were determined by comparing the fold increase in NF-κB induction by GMMA samples to the standard curve of NF-κB induction as a function of the FliC concentration. Flagellin bound in intact flagella does not stimulate TLR5 (41) and thus is not measured by this assay. Therefore, we refer to the flagellin content of GMMA measured by the TLR5-specific assay as “FliC equivalents”—specifically, equivalents to FliC from *S.* Typhimurium strain ATCC 14028, expressed in micrograms of FliC equivalents per milligram of GMMA protein. The specificity of the assay when used with the GMMA matrix was confirmed using GMMA from Shigella *sonnei*, since Shigella spp. do not produce flagella.

### Spiking experiments with FliC.

GMMA from STm_G_ Δ*msbB* Δ*pagP* and SEn_G_ Δ*msbB* Δ*pagP* (Table 1) were spiked with purified FliC from *S.* Typhimurium strain ATCC 14028 (Adipogen) at different concentrations (10 μg/mg GMMA [1% spike], 100 μg/mg GMMA [10% spike], and 1,000 μg/mg GMMA [100% spike]) and were analyzed by the TLR5-specific assay or the MAT in comparison to nonspiked GMMA and pure FliC.

### Statistical analysis.

Statistical analyses were performed using GraphPad Prism, version 6. All analyses were performed as two-tailed analyses. The nonparametric Kruskal-Wallis test followed by a *post hoc* Dunn multiple-comparison test was used to evaluate results from three or more different types of GMMA or treatments (TLR blocking, flagellin spiking) in the same assay. The nonparametric Mann-Whitney test was used to compare results for STm_G_ Δ*msbB* Δ*pagP* and SEn_G_ Δ*msbB* Δ*pagP* GMMA.

## RESULTS

### Characterization of GMMA-producing strains.

**FIG 1:**
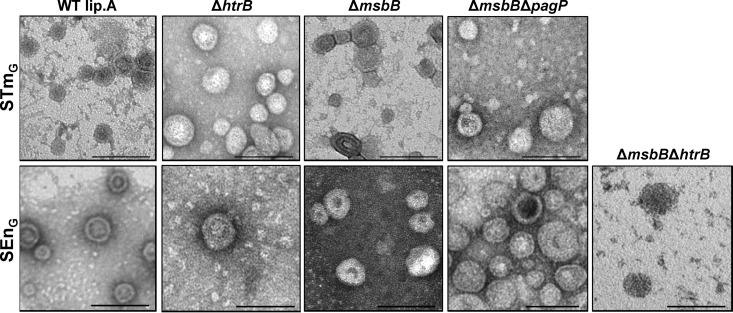
Electron microscopy of GMMA from different mutants. GMMA were purified from STm_G_ and SEn_G_ containing wild-type lipid A (WT lip.A), as well as from STm_G_ Δ*htrB*, STm_G_ Δ*msbB*, STm_G_ Δ*msbB* Δ*pagP*, SEn_G_ Δ*htrB*, SEn_G_ Δ*msbB*, SEn_G_ Δ*msbB* Δ*pagP*, and SEn_G_ Δ*msbB* Δ*htrB*. Magnification, ×87,000. Bars, 100 nm.

All Salmonella mutant strains (Table 1) were able to reach high ODs (OD 5 to 10) after overnight incubation at 30°C in LBON and yielded more than 50 mg GMMA protein/liter. The duplication times of the GMMA-producing *S.* Typhimurium and *S.* Enteritidis strains carrying the Δ*tolR* mutation (abbreviated as STm_G_ and SEn_G_, respectively) were approximately 30 min. In strains carrying lipid A modifications (the Δ*htrB*, Δ*msbB*, Δ*msbB* Δ*pagP*, and Δ*msbB* Δ*htrB* strains), the duplication times increased to 1 to 2 h. GMMA released from the different strains were evaluated by electron microscopy (Fig. 1). The size distributions and mean diameters of GMMA were determined by measuring 20 GMMA per strain and are listed in Table 3. The sizes of GMMA from the different strains were not significantly different (*P* = 0.39).

**TABLE 3:** GMMA characterization^*a*^

Strain	GMMA diam (nm)^*b*^		LPS content (nmol/mg GMMA)^*c*^	FliC equivalents (μg/mg GMMA)^*d*^
	Range (min–max)	Mean		
STm_G_	27–80	47	173	0.55
STm_G_ Δ*htrB*	27–87	39	165	0.74
STm_G_ Δ*msbB*	20–60	42	155	0.65
STm_G_ Δ*msbB* Δ*pagP*	27–107	51	160	0.22
SEn_G_	27–73	45	157	6.99
SEn_G_ Δ*htrB*	27–107	47	100	1.35
SEn_G_ Δ*msbB*	33–80	47	164	1.49
SEn_G_ Δ*msbB* Δ*pagP*	27–80	44	529	3.69
SEn_G_ Δ*msbB* Δ*htrB*	27–73	40	154	1.44

a Performed with single batches of GMMA purified from the different mutants.

b For 20 GMMA per type. min, minimum; max, maximum.

c Measured in duplicate per batch by quantification of KDO, with a coefficient of variance between the duplicate measurements of <5%.

d Monomeric flagellin was quantified using the TLR5-specific assay in comparison to a FliC standard and was expressed as FliC equivalents. The coefficient of variance for 4 independent experiments was approximately 30%. Means are shown.

### Characterization of lipid A.

**FIG 2:**
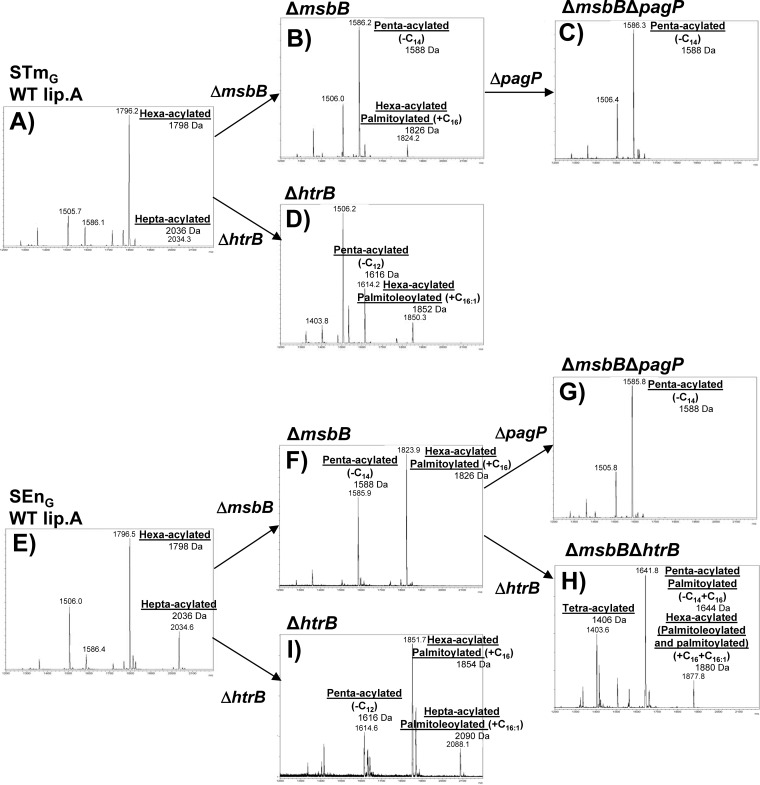
MALDI-TOF spectra of lipid A extracted from different GMMA. (A) STm_G_; (B) STm_G_ Δ*msbB*; (C) STm_G_ Δ*msbB* Δ*pagP*; (D) STm_G_ Δ*htrB*; (E) SEn_G_; (F) SEn_G_ Δ*msbB*; (G) SEn_G_ Δ*msbB* Δ*pagP*; (H) SEn_G_ Δ*msbB* Δ*htrB*; (I) SEn_G_ Δ*htrB*.

Lipid A was purified from GMMA from the various mutants and was analyzed by MALDI-TOF mass spectrometry (Fig. 2). Structures of lipid A corresponding to the main peaks were assigned based on mass and by comparison with results from similar Salmonella mutants (26). An overview is presented in Fig. S1 in the supplemental material. As expected, in the mass spectra of lipid A purified from GMMA from STm_G_ (Fig. 2A) and SEn_G_ (Fig. 2E), peaks were observed with *m/z* corresponding to the theoretical masses of hexa-acylated lipid A (1,798 Da) and hepta-acylated lipid A (2,036 Da), containing the additional palmitoyl fatty acid chain (C_16_; 238 Da) in the secondary position of myristoyl acid 2. In STm_G_, only a small amount of hepta-acylated lipid A was detected, whereas in SEn_G_, the intensity of the peak of the hepta-acylated lipid A was approximately 30% of the intensity of the peak of the hexa-acylated species.

Deletion of the *msbB* gene in both STm_G_ and SEn_G_ (Fig. 2B and F) resulted in lipid A species with *m/z* consistent with the theoretical masses of a penta-acylated lipid A (1,588 Da) and a hexa-acylated lipid A (1,826 Da), lacking the myristoyl chain (*m/z* shift of 210) compared to the WT hexa- or hepta-acylated lipid A, in line with the absence of MsbB. In SEn_G_ Δ*msbB* GMMA, the hexa-acylated lipid A was dominant. In contrast, in STm_G_ Δ*msbB* GMMA, the penta-acylated species was the most abundant, and very little hexa-acylated lipid A was detected, corresponding to the distribution of hexa- and hepta-acylated lipid A in GMMA from STm_G_ and SEn_G_ without lipid A modification.

After the deletion of the *pagP* gene in the Δ*msbB* mutants, penta-acylated lipid A (1,588 Da) was consistently the lipid A species with the highest molecular mass in both STm_G_ Δ*msbB* Δ*pagP* (Fig. 2C) and SEn_G_ Δ*msbB* Δ*pagP* (Fig. 2G), in line with the lack of palmitoylation of lipid A in the absence of PagP.

Deletion of *htrB* in STm_G_, SEn_G_, and SEn_G_ Δ*msbB* (Fig. 2D, I, and H) resulted in a variety of lipid A forms. Lipid A species consistent with the lack of the lauroyl chain (*m/z* decrease of 182) from the lipid A species identified in the respective parent strain were observed in all Δ*htrB* GMMA, i.e., 1,616-Da penta-acylated lipid A in STm_G_ Δ*htrB* and SEn_G_ Δ*htrB*, 1,854-Da hexa-acylated palmitoylated lipid A in SEn_G_ Δ*htrB*, and 1,406-Da tetra-acylated and 1,644-Da palmitoylated, penta-acylated lipid A in SEn_G_ Δ*msbB* Δ*htrB*. In addition, replacement of the lauroyl chain (C_12_; 182 Da) with a palmitoleoyl chain (C_16:1_; 236 Da) was observed as expected (20, 24, 28) and was consistent with the activity of the late acyl transferase LpxP. These lipid A species included hepta-acylated palmitoleoylated lipid A (2,090 Da) in SEn_G_ Δ*htrB* GMMA (Fig. 2I) and hexa-acylated palmitoleoylated lipid A (1,852 Da) in STm_G_ Δ*htrB* GMMA (Fig. 2D), in accordance with the distribution of hexa- and hepta-acylated lipid A in GMMA from STm_G_ and SEn_G_ (Fig. 2A and E), and 1,880-Da palmitoylated and palmitoleoylated lipid A in GMMA from SEn_G_ Δ*msbB* Δ*htrB*. In consideration of these pleiotropic effects, no STm_G_ Δ*msbB* Δ*htrB* mutant was generated.

In order to assess whether the different lipid A structures have an impact on the total content of lipid A in the different GMMA, we determined the molar amount of LPS per milligram of protein by quantifying the LPS core sugar KDO (Table 3). For most GMMA, the molar LPS contents were similar, with approximately 160 nmol/mg GMMA. Surprisingly, in GMMA from SEn_G_ Δ*msbB* Δ*pagP*, the LPS content was more than twice as high (529 nmol/mg GMMA). To verify the high LPS content in GMMA from SEn_G_ Δ*msbB* Δ*pagP*, two other batches were prepared and measured. The respective LPS contents were 310 and 428 nmol LPS/mg GMMA.

### Cytokine release from human PBMC.

**FIG 3:**
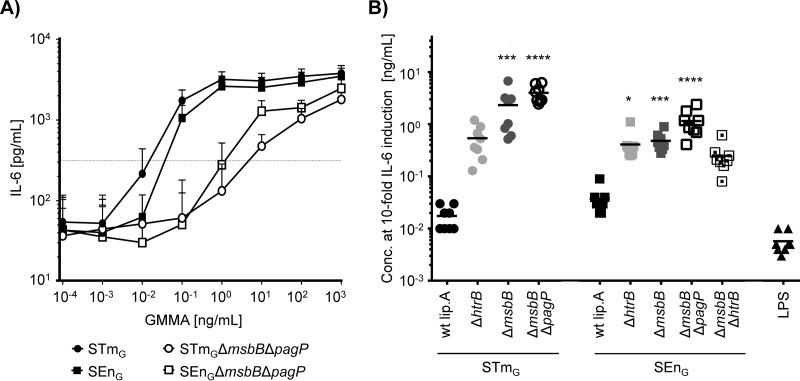
IL-6 release by human PBMC after stimulation with GMMA. Human PBMC were stimulated with GMMA, and IL-6 release was measured. (A) The average levels of IL-6 release in response to GMMA with unmodified lipid A (STm_G_, SEn_G_) and in response to GMMA with solely penta-acylated lipid A (STm_G_ Δ*msbB* Δ*pagP*, SEn_G_ Δ*msbB* Δ*pagP*) are plotted against the GMMA concentration. Error bars show standard deviations for 8 independent experiments using PBMC from 4 different donors. The dashed line indicates the IL-6 concentration at 10-fold over background. (B) Scatter plot of GMMA concentrations resulting in a 10-fold increase in IL-6 release. GMMA from STm_G_, STm_G_ Δ*msbB*, STm_G_ Δ*msbB* Δ*pagP*, STm_G_ Δ*htrB*, SEn_G_, SEn_G_ Δ*msbB*, SEn_G_ Δ*msbB* Δ*pagP*, SEn_G_ Δ*msbB* Δ*htrB*, and SEn_G_ Δ*htrB* were analyzed. LPS from E. coli was used as s benchmark control. The results for GMMA from *S.* Typhimurium and *S.* Enteritidis were compared using the Kruskal-Wallis test with Dunn's *post hoc* multiple comparisons. Asterisks indicate GMMA with lipid A modification that gave results statistically different from those for GMMA with unmodified lipid A (*, *P* ≤ 0.05; ***, *P* ≤ 0.001; ****, *P* ≤ 0.0001).

We assessed the potential of the STm_G_ and SEn_G_ GMMA with different lipid A compositions to stimulate innate immune responses by characterizing their potential to induce IL-6 release from human PBMC using a monocyte activation test (MAT). IL-6 was chosen because in our recent studies with Shigella GMMA, IL-6 release was representative of the release of proinflammatory cytokines, including TNF-α, IL-1β, IL-8, IL-12p70, and gamma interferon (IFN-γ) (20). The stimulatory activities of the GMMA were compared based on the concentration required to elicit a 10-fold increase in IL-6 release over background (Fig. 3), as reported previously (20). STm_G_ and SEn_G_ GMMA without lipid A modifications induced a 10-fold increase in IL-6 release at low concentrations. These GMMA concentrations were, on average, only 3 times (STm_G_) or 7 times (SEn_G_) higher than the concentration of purified E. coli LPS required to induce a 10-fold increase. In contrast, substantially higher concentrations of GMMA with lipid A modifications were needed to induce IL-6 release (Fig. 3). For both *S.* Typhimurium and *S.* Enteritidis, GMMA from the Δ*msbB* Δ*pagP* mutants showed the most significant differences from GMMA without lipid A modification. The amount of STm_G_ Δ*msbB* Δ*pagP* GMMA required to induce a 10-fold increase in IL-6 release was approximately 200-fold higher than the amount of STm_G_ GMMA with WT lipid A. For the respective *S.* Enteritidis GMMA, the difference was approximately 30-fold. Interestingly, the concentrations of GMMA with purely penta-acylated lipid A from the Δ*msbB* Δ*pagP* mutants of *S.* Enteritidis and *S.* Typhimurium that were required to elicit a 10-fold increase in IL-6 release from human PBMC showed a 3-fold difference (mean for SEn_G_ Δ*msbB* Δ*pagP* GMMA, 1.17 ng/ml; mean for STm_G_ Δ*msbB* Δ*pagP* GMMA, 4.02 ng/ml) that was statistically significant (*P* = 0.0002).

### Quantification of monomeric flagellin in GMMA using a TLR5-specific assay.

Since flagellin impurities could potentially contribute to the differences in activity between different GMMA, we quantified the flagellin contents in the samples. Taking advantage of the fact that flagellin specifically stimulates TLR5 (19, 41), we used human embryonic kidney (HEK293) cells expressing human TLR5 and an NF-κB-inducible luciferase reporter gene, and we determined the concentration of flagellin in GMMA in comparison to a standard curve generated with purified FliC, the major Salmonella flagellin. Because the assay is specific for monomeric flagellin, we refer to the measured flagellin concentrations as FliC equivalents. The concentrations of FliC equivalents in the different GMMA ranged from 0.22 to 6.99 μg/mg GMMA protein (Table 3). Interestingly, the FliC equivalent contents differed statistically (*P* = 0.016) between GMMA from the STm_G_ strains (mean of FliC equivalents in the 4 different types, 0.54 μg/mg GMMA) and GMMA from the SEn_G_ strains (mean, 2.99 μg/mg GMMA).

### Contributions of individual TLRs to GMMA stimulation.

**FIG 4:**
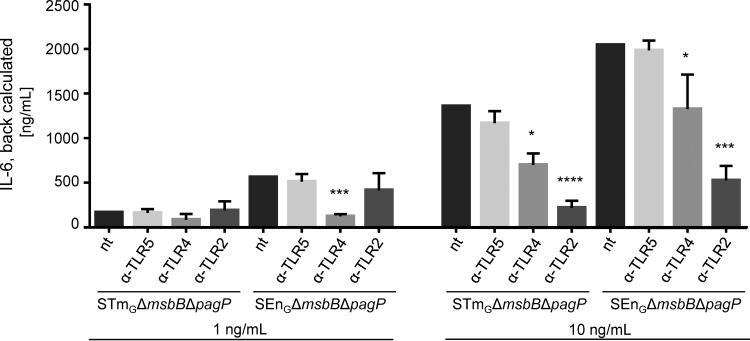
Effect of TLR blocking on IL-6 stimulation by GMMA from STm_G_ Δ*msbB* Δ*pagP* or SEn_G_ Δ*msbB* Δ*pagP*. Human PBMC either were incubated with anti-TLR5, anti-TLR4, or anti-TLR2 or were left untreated (not treated [nt]) and were subsequently stimulated with 1 ng/ml or 10 ng/ml of GMMA. Results in each TLR-blocking experiment were normalized according to the results for untreated PMBC in the same experiment. To illustrate the different levels of stimulation by the different GMMA and concentrations, the normalized results were then back calculated to the average IL-6 production in the untreated samples. Error bars show standard deviations with 6 independent replicates using PBMC from 3 different donors. The results for untreated PBMC and for TLR2-, TLR4-, and TLR5-blocked PBMC using the same type and concentration of GMMA were compared using the Kruskal-Wallis test with Dunn's *post hoc* multiple comparisons. Asterisks indicate treatments that gave results statistically different from those for the untreated samples (*, *P* ≤ 0.05; ***, *P* ≤ 0.001; ****, *P* ≤ 0.0001).

To investigate whether the 3-fold difference in stimulatory potential between GMMA from SEn_G_ Δ*msbB* Δ*pagP* and GMMA from STm_G_ Δ*msbB* Δ*pagP* is linked to the stimulation of a specific TLR, we determined the contributions of individual TLRs to the residual activation of cytokine release using TLR-blocking and TLR-specific activation assays. For TLR-blocking experiments, PBMC were incubated with TLR2-, TLR4-, or TLR5-blocking antibodies before stimulation with 1 or 10 ng of GMMA/ml, concentrations chosen to give significant increases in IL-6 in the range of the linear part of the curves or just reaching saturating conditions at the upper plateau of the sigmoidal response curve (Fig. 3). The results of the TLR-blocking experiments were similar for the two types of GMMA (Fig. 4): at 10 ng of GMMA/ml, IL-6 production was significantly reduced following incubation with a TLR2-blocking antibody (mean remaining activity, 16% for STm_G_ and 26% for SEn_G_) or a TLR4-blocking antibody (mean remaining activity, 52% for STm_G_ and 65% for SEn_G_). In contrast, blocking TLR5 did not result in a statistically significant reduction in IL-6 production (mean remaining activity, 86% for STm_G_ and 97% for SEn_G_). At the 1-ng/ml concentration, the level of stimulation with GMMA from STm_G_ Δ*msbB* Δ*pagP* was very low, and no significant differences were observed after blocking TLR2, TLR4, or TLR5. The 1-ng/ml concentration of SEn_G_ Δ*msbB* Δ*pagP* GMMA elicited higher levels of IL-6 than the same concentration of STm_G_ Δ*msbB* Δ*pagP*, and blocking with TLR4 significantly reduced IL-6 release.

**FIG 5:**
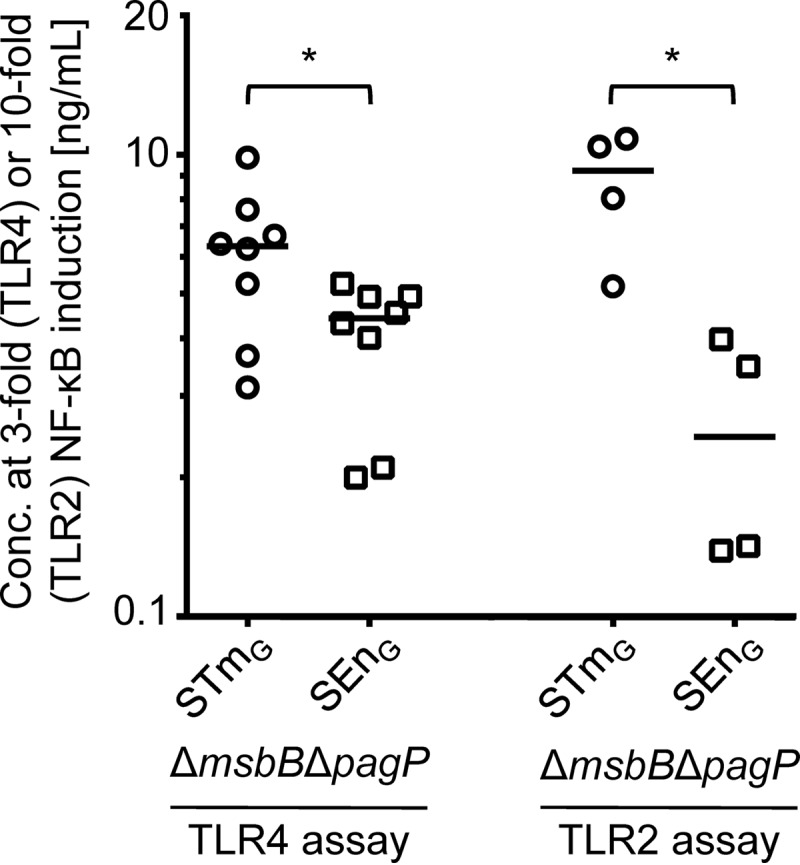
TLR4 and TLR2 activation by GMMA from Δ*msbB* Δ*pagP* mutants of STm_G_ and SEn_G_. The concentrations of STm_G_ Δ*msbB* Δ*pagP* GMMA and SEn_G_ Δ*msbB* Δ*pagP* GMMA resulting in a 3-fold increase in NF-κB activity in HEK-TLR4 cells or a 10-fold increase in NF-κB activity in HEK-TLR2 cells are shown. Results were analyzed using the Mann-Whitney test.

Since the contributions of the different TLRs to PBMC stimulation were similar for STm_G_ Δ*msbB* Δ*pagP* and SEn_G_ Δ*msbB* Δ*pagP* GMMA, based on the blocking experiments, we further investigated the potential of the GMMA to stimulate TLR4 and TLR2 in specific assays. As shown in Fig. 5, both assays showed trends equivalent to those observed before, with SEn_G_ Δ*msbB* Δ*pagP* GMMA eliciting a slightly (approximately 1.5-fold in the TLR4 assay and approximately 3.4-fold in the TLR2 assay) but statistically significantly higher level of stimulation than STm_G_ Δ*msbB* Δ*pagP*. SDS-PAGE analysis (see Fig. S2 in the supplemental material) showed that the amounts of protein in the preparations were similar but that the protein patterns were quite different. Whether the different protein compositions are related to the difference in TLR2 activation remains to be determined.

### FliC spiking experiments.

**FIG 6:**
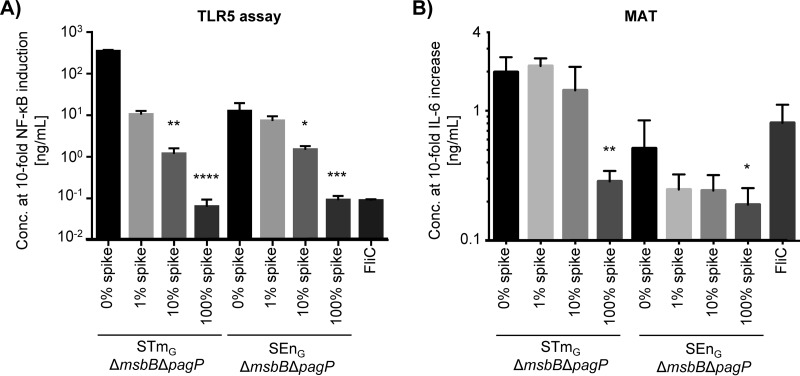
Impact of FliC spiking of GMMA in the TLR5-specific assay (A) or the MAT (B). GMMA from STm_G_ Δ*msbB* Δ*pagP* and SEn_G_ Δ*msbB* Δ*pagP* were spiked with FliC at different concentrations—10 μg/mg GMMA (1% spike), 100 μg/mg GMMA (10% spike), and 1,000 μg/mg GMMA (100% spike)—and were analyzed by a TLR5-specific assay or by the MAT in comparison to the respective nonspiked GMMA (0% spike) and pure FliC. Sample concentrations (for spiked GMMA corresponding to the concentration of GMMA in the sample) resulting in 10-fold-increased activation in the respective assay were determined in 6 independent experiments. Error bars show standard deviations. The results were analyzed using the Kruskal-Wallis test with Dunn's *post hoc* multiple comparison. Asterisks indicate spiked samples that gave results statistically different from those for the corresponding nonspiked GMMA (*, *P* ≤ 0.05; **, *P* ≤ 0.01; ***, *P* ≤ 0.001; ****, *P* ≤ 0.0001).

In the TLR-blocking experiments, TLR5 activation by GMMA did not contribute significantly to the observed IL-6 release. This suggested that the monomeric flagellin level found in the GMMA tested is likely in an acceptable range. For further development of Salmonella GMMA as vaccines, we wanted to understand if there is a threshold concentration of monomeric flagellin above which TLR5 activation will significantly increase the stimulatory potential of GMMA. To test this, we spiked GMMA from Δ*msbB* Δ*pagP* mutants of STm_G_ and SEn_G_ with purified FliC at three different concentrations (spike 1, 10 μg FliC/mg GMMA [1%]; spike 2, 100 μg FliC/mg GMMA [10%]; spike 3, 1,000 μg FliC/mg GMMA [100%]), assessed activity using the TLR5-specific assay and the MAT, and compared the results with those for GMMA alone and a FliC standard (Fig. 6). In the TLR5-specific assay, the increase in activity was proportional to the spiked amount of FliC (Fig. 6A), and the concentrations of FliC equivalents found in the GMMA samples in comparison to the FliC standard were within the expected range. The concentrations of FliC equivalents determined with the 0%, 1%, 10%, and 100% FliC spikes were 0.3, 10, 113, and 1,460 μg/mg GMMA protein in STm_G_ Δ*msbB* Δ*pagP* GMMA and 4, 14, 104, and 1,004 μg/mg GMMA protein in SEn_G_ Δ*msbB* Δ*pagP* GMMA. Thus, no interference of the GMMA matrix with the spiked FliC was detected. In contrast, in the MAT, the spiking of GMMA with FliC had surprisingly little effect on IL-6 release (Fig. 6). For both STm_G_ Δ*msbB* Δ*pagP* and SEn_G_ Δ*msbB* Δ*pagP* GMMA, only spiking with 1,000 μg of FliC/mg GMMA (100%) resulted in a statistically significant increase in IL-6 stimulation (Fig. 6).

## DISCUSSION

The goal of this study was to identify the most promising approach for reducing the potential reactogenicity of an iNTS GMMA vaccine for use in humans against *S.* Typhimurium and *S.* Enteritidis by lipid A modification and to assess the impact of flagellin as a potential reactogenic impurity. As in previous studies (20, 39), cytokine release from human cells, and particularly IL-6 release (12, 20), was chosen as an *in vitro* readout of potential reactogenicity.

Although the impacts of lipid A modification in Salmonella Typhimurium on the reactogenicity and immunogenicity of LPS and bacteria have been widely studied (24–27, 29), the availability of GMMA from isogenic mutants enabled us to compare such effects in *S.* Typhimurium and the less-studied organism *S.* Enteritidis. Deletion of *htrB* gave a pleiotropic outcome, as described previously (24), including replacement of the lauroyl chain by a palmitoleoyl chain that was also found in *htrB* mutants of E. coli and Shigella flexneri 2a (20, 28). Due to the complex outcome, the differences in composition between the *S.* Typhimurium and *S.* Enteritidis mutants, and the higher stimulatory potential of GMMA from Δ*htrB* mutants, including SEn_G_ Δ*msbB* Δ*htrB*, deletion of *htrB* was not pursued. Deletion of *msbB* alone in GMMA-producing strains (26) gave a mixture of penta-acylated and palmitoylated hexa-acylated lipid A, and deletion of both *msbB* and *pagP* gave only penta-acylated lipid A, as expected. The distribution of the penta- and palmitoylated hexa-acylated lipid A in the *msbB* mutants corresponded to the observed distribution of hexa- and hepta-acylated lipid A in GMMA from the parent strain without lipid A modification.

While MALDI-TOF analysis is semiquantitative, the results of the MAT were consistent with the observed distribution of lipid A species: GMMA containing hexa-acylated or hepta-acylated lipid A alone or in addition to penta-acylated lipid A resulted in more activation, whereas GMMA with predominantly or purely penta-acylated lipid A gave minimal activation. Penta-acylated lipid A has been reported to have antagonistic activity on human TLR4 and to inhibit activation by hexa-acylated lipid A (42). However, the higher stimulatory potential of GMMA with a mixture of penta-acylated and palmitoylated hexa-acylated lipid A suggested that the hexa-acylated lipid A was recognized by TLR4. Whether penta-acylated lipid A presented in the context of LPS in GMMA is or is not available to act as an antagonist and whether the activity of palmitoylated hexa-acylated lipid A is or is not blocked by penta-acylated lipid A will be the subjects of future studies.

GMMA from the Δ*msbB* Δ*pagP* mutants were chosen for further vaccine development, based on the uniformly penta-acylated lipid A and the lowest stimulatory potential in the MAT. The stimulatory potentials to elicit a 10-fold increase in IL-6 release from PBMC (for STm_G_ Δ*msbB* Δ*pagP* GMMA, the average concentration required for such an increase from all 14 independent experiments [Fig. 3 and 6] was 3.15 ng/ml, and for SEn_G_ Δ*msbB* Δ*pagP*, it was 0.89 ng/ml) were similar to that of a Shigella sonnei GMMA vaccine candidate currently in phase I clinical trials (2.37 ng/ml [12]). The decrease in stimulatory potential from that of GMMA with WT lipid A was approximately 200-fold for STm_G_ Δ*msbB* Δ*pagP* GMMA and approximately 30-fold for SEn_G_ Δ*msbB* Δ*pagP* GMMA. While the level of reduction of GMMA reactogenicity required for an acceptable vaccine will depend on the dose required to give a strong immune response, which can be determined only in clinical trials, these data suggest that lipid A modification through deletion of *msbB* and *pagP* is a promising strategy for minimizing the reactogenicity of an iNTS GMMA vaccine.

The residual activity of GMMA from STm_G_ Δ*msbB* Δ*pagP* and SEn_G_ Δ*msbB* Δ*pagP* was due largely to TLR2 activation. At the same time, these GMMA still signaled significantly through TLR4, a finding similar to that with Shigella Δ*msbB* GMMA (20). This might be a special feature of Δ*msbB* GMMA with penta-acylated lipid A lacking the myristoyl chain compared to hexa-acylated lipid A, since, for example, GMMA from Shigella sonnei Δ*htrB* containing penta-acylated lipid A lacking the lauroyl chain did not stimulate TRL4 (20). Interestingly, although GMMA from both STm_G_ Δ*msbB* Δ*pagP* and SEn_G_ Δ*msbB* Δ*pagP* contained only penta-acylated lipid A, they showed an approximately 3-fold difference in their potentials to elicit IL-6 release from human PBMC (3.15 ng/ml versus 0.89 ng/ml), which was highly significant (*P* < 0.0001). While the relative contributions of TLR2 and TLR4 stimulation were similar, GMMA from SEn_G_ Δ*msbB* Δ*pagP* showed a higher stimulatory potential for TLR4 as well as for TRL2. Higher activation of TLR4 is likely due in part to the 3-fold-higher LPS content. The reasons for this higher LPS content are unclear. The higher TLR2 activation could be related to different protein compositions as visualized by SDS-PAGE. The identities of the proteins present in the different visible bands and the question of whether these proteins are likely to result in TLR2 activation remain to be determined. Also, penta-acylated forms of LPS have been shown to act as TLR2 agonists (43). Therefore, we cannot exclude the possibility that the penta-acylated lipid A in GMMA could contribute to residual TLR2 activity and that the 3-fold-higher LPS content in SEn_G_ Δ*msbB* Δ*pagP* GMMA could also be linked to the 3-fold-higher TLR2 stimulation.

A potential source of reactogenicity in Salmonella GMMA that is absent in Shigella GMMA is the TLR5 activator flagellin. GMMA purified from STm_G_ Δ*msbB* Δ*pagP* and SEn_G_ Δ*msbB* Δ*pagP* contained only a small amount of monomeric flagellin as evaluated by the TRL5-specific assay (0.2 to 7.0 μg FliC equivalents/mg GMMA protein). No interference of the GMMA matrix was detected in the TLR5-specific assay, and thus, this assay provides a convenient tool for quantifying monomeric flagellin in samples. However, intact flagella do not stimulate TLR5 and thus are not detected in the TRL5-specific assay (41). Because flagellin monomers might be shed from potentially present flagella, additional assays will be required to measure total flagellin in GMMA samples.

Interestingly, spiking GMMA with increasing concentrations of FliC up to 10% of total GMMA protein did not significantly increase the stimulation of PBMC in the MAT. Similarly, no significant difference in GMMA-mediated stimulation was observed by the MAT when TLR5 was blocked. Together, these results suggest that monomeric flagellin impurities in GMMA, at the concentrations observed in this study, do not contribute significantly to GMMA-mediated IL-6 release from PBMC, at least *in vitro*. This result was in contrast to findings with Pseudomonas aeruginosa NOMV in which flagellin contributed significantly to IL-6 stimulation from mouse macrophages (17). The different outcomes might be related to the use of human PBMC as opposed to the mouse MH-S macrophage cell line or to a potentially higher flagellin content in P. aeruginosa NOMV, since the flagellin gave a visible band by SDS-PAGE (17). Alternatively, they may suggest that the MAT, or particularly the release of IL-6, under the conditions we used is not sufficiently sensitive to measure flagellin-mediated responses.

Thus, we compared the level of FliC equivalents measured in GMMA with the flagellin content of a flagellin-adjuvanted vaccine candidate. A 100-μg dose of Shigella GMMA formulated with Alhydrogel (1790GAHB) administered intramuscularly was found to be well tolerated and immunogenic in rabbits in a repeat-dose toxicity study and is currently being evaluated as the highest dose in a phase I dose-escalation clinical trial (12). A 100-μg dose of iNTS GMMA would correspond to 0.37 μg FliC equivalents/dose for SEn_G_ Δ*msbB* Δ*pagP* GMMA and to 0.02 μg FliC equivalents/dose for STm_G_ Δ*msbB* Δ*pagP* GMMA. VAX128C, a recombinant influenza virus-hemagglutinin-flagellin fusion vaccine with a flagellin content of 46%, was found in a phase I clinical trial to be well tolerated at doses as high as 20 μg (corresponding to 9.2 μg flagellin) and highly immunogenic at doses of 1.25 to 2.5 μg (0.58 to 1.15 μg of flagellin) (44). With the caveat that the total flagellin content remains to be measured, a 100-μg dose of iNTS GMMA contains less monomeric flagellin (in FliC equivalents) than is present in the lowest dose of VAX128C.

In this study, we used solely IL-6 release as an indicator for proinflammatory responses to TLR stimulation, based on our previous study with Shigella GMMA (20). Since Shigella spp. lack flagellin, this is a limitation of this study. Future analysis of additional proinflammatory and immunomodulating cytokines might shed additional light on the contribution of flagellin and the remaining stimulatory capacity of iNTS Δ*msbB* Δ*pagP* GMMA. Still, we think that the significant reduction in the stimulatory potential observed in the IL-6 data provides a strong rationale for proceeding to *in vivo* studies in rabbits as the next step in the evaluation of the safety of iNTS GMMA.

In conclusion, the data indicate that deletion of *msbB* and *pagP* is a promising approach to minimizing the reactogenicity of iNTS GMMA vaccines for use in humans, and this approach has been selected for further development. Also, flagellin impurities are likely at an acceptable level but require additional evaluation. With the scale-up of production and the use of tangential flow filtration for the purification of GMMA (10, 12), it will be important to assess whether the purification method has an impact on the presence of monomeric and total flagellin and the stimulatory potential of GMMA *in vitro* as well as *in vivo*.

## Supplementary Material

Supplemental material
