# Supplementary material for: Toll-Like Receptor Activation by Generalized Modules for Membrane Antigens from Lipid A Mutants of Salmonella enterica Serovars Typhimurium and Enteritidis
Source: Clin Vaccine Immunol. 2016 Apr 4;23(4):304–14. doi: 10.1128/CVI.00023-16 (PMC4820502; doi:10.1128/CVI.00023-16)
Supplement: Supplemental material [file supp_23_4_304__index.html]

Toll-Like Receptor Activation by Generalized Modules for Membrane Antigens from Lipid A Mutants of Salmonella enterica Serovars Typhimurium and Enteritidis — Supplemental material 

# Toll-Like Receptor Activation by Generalized Modules for Membrane Antigens from Lipid A Mutants of Salmonella enterica Serovars Typhimurium and Enteritidis

## Supplemental material

- Supplemental file 1 -

  Fig. S1. Scheme of expected lipid A species in the different mutants, originating from hepta-acylated lipid A. Fig. S2. SDS-PAGE protein profile of GMMA from STmGΔ*msbB*Δ*pagP* and SEnGΔ*msbB*Δ*pagP*.

  PDF, 425K
