# Supplementary material for: Toll-Like Receptor Activation by Generalized Modules for Membrane Antigens from Lipid A Mutants of Salmonella enterica Serovars Typhimurium and Enteritidis
Source: Clin Vaccine Immunol. 2016 Apr 4;23(4):304–14. doi: 10.1128/CVI.00023-16 (PMC4820502; doi:10.1128/CVI.00023-16)
Supplement: Supplemental material [file CVI.00023-16_zcd999095332so1.pdf]

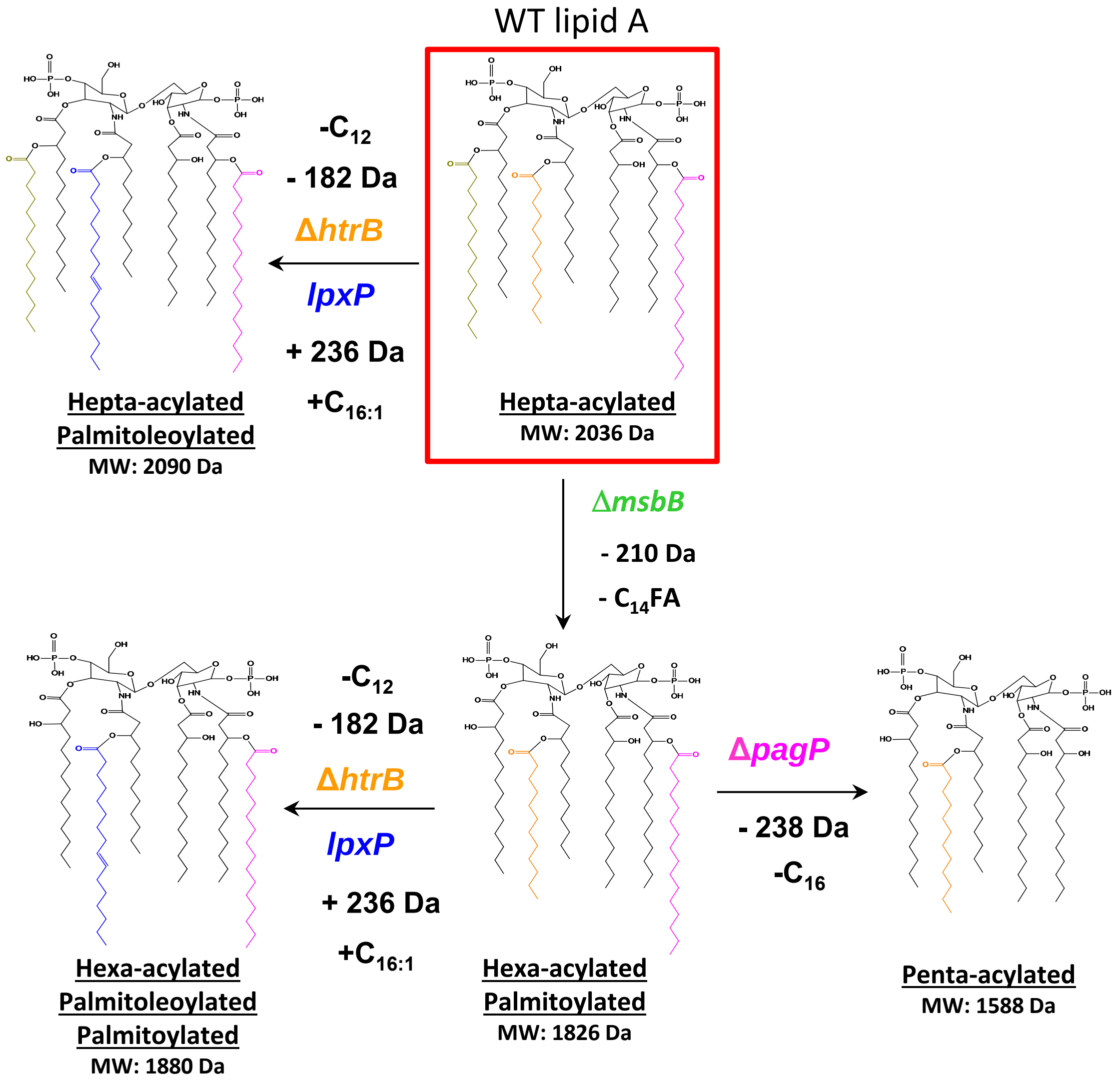

FIGURE 1S. Scheme of expected lipid A species in the different mutants, originating from hepta-acylated lipid A.

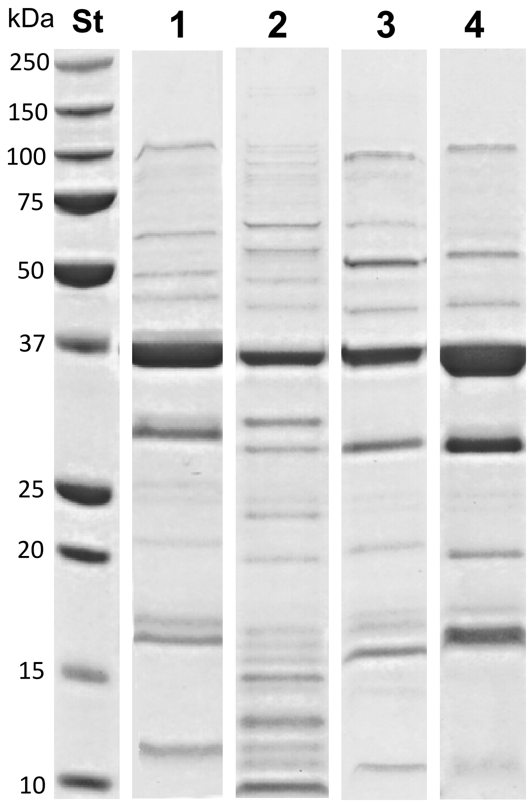

Figure 2S. SDS-PAGE protein profile of GMMA from STm$_G$Δ*msbB*Δ*pagP* (lane 2) and SEn$_G$Δ*msbB*Δ*pagP* (lane 3). 10 µg (GMMA protein) were loaded on a 12% SDS-PAGE. For comparison, the protein patterns of GMMA from STm$_G$ (lane 1) and SEn$_G$ (lane 4) without lipid A modification are shown.
